# Subcutaneous Section of Adhesions for Reduction of Old Dislocations of the Shoulder-joint

**Published:** 1882-07

**Authors:** 


### SUBCUTANEOUS SECTION OF ADHESIONS FOR REDUCTION OF OLD DISLOCATIONS OF THE SHOULDER-JOINT.

The fibrous adherences which form around the displaced head of the humerus are the great obstacle to be overcome in reducing the luxation. After six months for sub-coracoid luxations, and four months for the intra-coracoid, they are generally so firm as to render reduction impossible. Fracture of the neck of the humerus has already been proposed by Després for these irreducible luxations. M. Polaillon advocates the subcutaneous division of the fibrous bands, and has been able by this means to reduce a luxation of four months' standing, and recommends that whenever an extending weight of 200 or 300 pounds is unable to displace the head of the bone, the adherences should be divided, antiseptic precautions being taken, after etherizing the subject. —*Revue de Chirurgie,* April 10, 1882.

